# QTL Analysis of Resistance to High-Intensity UV-B Irradiation in Soybean (*Glycine max* [L.] Merr.)

**DOI:** 10.3390/ijms20133287

**Published:** 2019-07-04

**Authors:** Min Young Yoon, Moon Young Kim, Jungmin Ha, Taeyoung Lee, Kyung Do Kim, Suk-Ha Lee

**Affiliations:** 1 Department of Plant Science and Research Institute of Agriculture and Life Sciences, Seoul National University, Seoul 08826, Korea; 2 Plant Genomics and Breeding Institute, Seoul National University, Seoul 08826, Korea; 3 Corporate R&D, LG Chem, Seoul 07795, Korea

**Keywords:** soybean, UV-B stress resistance, quantitative trait loci, spectrin beta chain, brain, bZIP transcription factor21/TGA9, leucine-rich repeat family protein, stress defense signaling

## Abstract

High-intensity ultraviolet-B (UV-B) irradiation is a complex abiotic stressor resulting in excessive light exposure, heat, and dehydration, thereby affecting crop yields. In the present study, we identified quantitative trait loci (QTLs) for resistance to high-intensity UV-B irradiation in soybean (*Glycine max* [L.]). We used a genotyping-by-sequencing approach using an F6 recombinant inbred line (RIL) population derived from a cross between Cheongja 3 (UV-B sensitive) and Buseok (UV-B resistant). We evaluated the degree of leaf damage by high-intensity UV-B radiation in the RIL population and identified four QTLs, *UVBR12-1*, *6-1*, *10-1*, and *14-1*, for UV-B stress resistance, together explaining 20% of the observed phenotypic variation. The genomic regions containing *UVBR12-1* and *UVBR6-1* and their syntenic blocks included other known biotic and abiotic stress-related QTLs. The QTL with the highest logarithm of odds (LOD) score of 3.76 was *UVBR12-1* on Chromosome 12, containing two genes encoding spectrin beta chain, brain (SPTBN, Glyma.12g088600) and bZIP transcription factor21/TGACG motif-binding 9 (bZIP TF21/TGA9, Glyma.12g088700). Their amino acid sequences did not differ between the mapping parents, but both genes were significantly upregulated by UV-B stress in Buseok but not in Cheongja 3. Among five genes in *UVBR6-1* on Chromosome 6, Glyma.06g319700 (encoding a leucine-rich repeat family protein) had two nonsynonymous single nucleotide polymorphisms differentiating the parental lines. Our findings offer powerful genetic resources for efficient and precise breeding programs aimed at developing resistant soybean cultivars to multiple stresses. Furthermore, functional validation of the candidate genes will improve our understanding of UV-B stress defense mechanisms.

## 1. Introduction

Increased solar ultraviolet-B radiation (UV-B, 280–315 nm) since the late 1980s is considered a serious environmental issue owing to the lengthy expected time of the recovery of the destroyed stratospheric ozone layer to pre-1980 levels [1,2]. High-intensity UV-B radiation beyond the level of positive stimulus to sessile plants exerts multiple stresses, such as strong light, high temperatures, and dehydration, causing physiological and morphological damages including reduced photosynthetic capacity, leaf discoloration, and reduced biomass and seed yields [3,4]. According to the United Nations Environment Programme (UNEP) annual report, increased UV-B radiation in terrestrial areas reduces plant productivity by about 6% [5]. To prevent such yield losses in crop plants, genetic studies of resistance to high-intensity UV-B radiation as a complex abiotic stress and the identification of genetic elements involved in the UV-B defense response are needed for major crops across the world.

Soybean (*Glycine max* [L.] Merr.) is one of the most important crops for food, feed, energy production, and industrial resources worldwide. Under supplemental UV-B radiation, soybean cultivars show differences in physiological damage and morphological changes; UV-B-sensitive cultivars show significant yield reductions [6,7,8]. Quantitative trait loci (QTLs) associated with resistance to supplementary UV-B treatment have been identified on chromosomes (Chrs) 3, 6, 7, and 19 using a recombinant inbred line (RIL) population derived from a cross between Keunol (UV-B sensitive) and Iksan10 (UV-B resistant) [9]. Using the same population, the QTL *qUVBT1* on Chr 7 was identified with the 180K AXIOM SoyaSNP array and a gene encoding a UV excision repair protein was identified as a candidate gene involved in UV-B tolerance [10]. In our previous study, we identified the most resistant and most sensitive genotypes to elevated UV-B, Buseok and Cheongja 3, among 140 soybean genotypes, including 94 *G. max* and 46 *G. soja* accessions [11]. Transcriptome profiling of these two genotypes differing in UV-B resistance has revealed differentially expressed genes involved in stress defense signaling, immune responses, and reactive oxygen species metabolism [12].

In the present study, we identified QTLs associated with resistance to high-intensity UV-B irradiation using an F_6_ RIL population of Cheongja 3 (UV-B sensitive) × Buseok (UV-B resistant). Furthermore, we investigated nucleotide variation in genes located in the QTLs in the mapping parents and their expression levels in response to UV-B treatment.

## 2. Results

### 2.1. Phenotypic Evaluation of UV-B Stress Resistance in a RIL Population of Cheongja 3 × Buseok

**Figure 1:**
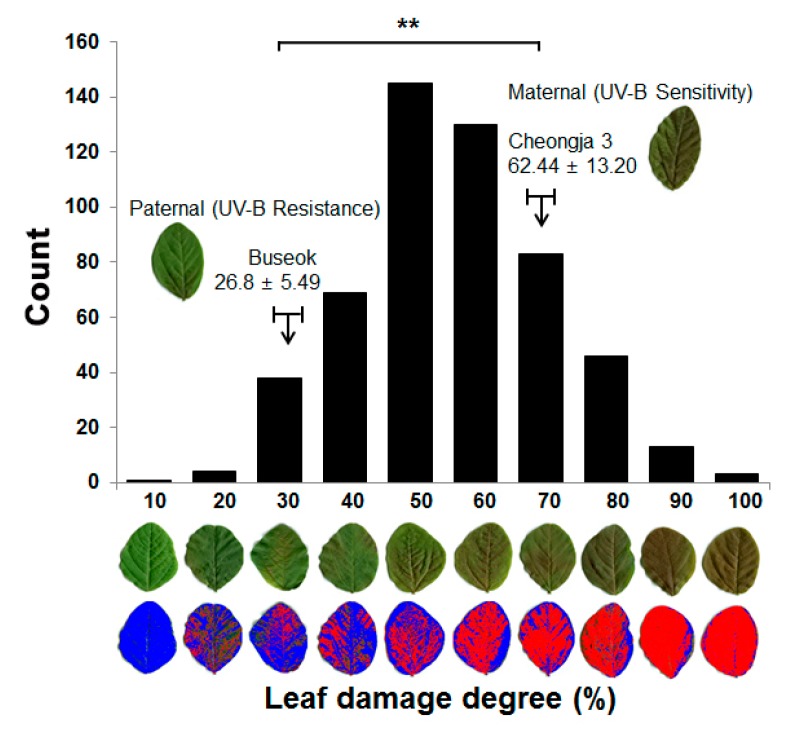
Distribution of the degree of leaf damage under high-intensity ultraviolet-B (UV-B) irradiation in the recombinant inbred line (RIL) population of Cheongja 3 × Buseok. X-axis indicates the degree of leaf damage from 10% to 100%. For each degree of damage, RGB (red-green-blue) and two-color-transformed images of a representative injured leaf are shown. Blue and red in the two-color-transformed image indicate intact and damaged parts of the leaf exposed to UV-B irradiation, respectively. ** indicates a significant difference between Cheongja 3 and Buseok at *p* < 0.01 based on a Student’s *t*-test.

UV-B-resistant Buseok and UV-B-sensitive Cheongja 3 showed leaf damage of 26.8% and 62.4%, respectively, in response to high-intensity UV-B irradiation (Figure 1). The difference in UV-B resistance between the two parents was consistent with the results of previous studies [11,12]. The RIL population of Cheongja 3 × Buseok showed high phenotypic variation in leaf damage, ranging from 10% to 100%, with a mean value of 50.3% (Figure 1). The normal distribution of the degree of UV-B leaf damage in the RIL population (Shapiro-Wilk: W = 0.993, P = 0.665), with transgressive segregation, indicates that UV-B resistance is quantitatively regulated by multiple genes.

### 2.2. Genotyping-by-Sequencing Analysis and Genetic Linkage Map Construction

Out of about 562 million genotyping-by-sequencing (GBS) reads generated from two libraries of the two parents and 174 RILs, 441,142,257 (78.4%) high-quality clean reads were obtained (Table S1). The number of reads per line ranged from 1,147,211 to 10,657,712 with an average of 3,197,053 reads. From these reads, we identified 271,254 unfiltered single nucleotide polymorphisms (SNPs) differentiating Cheongja 3 and Buseok. We used 4604 high-quality single nucleotide polymorphisms after filtering to construct a genetic linkage map of the RIL population. The final linkage map contained 3136 SNP markers evenly distributed on 20 chromosomes according to their physical locations on the soybean reference genome (Figure S1 and Table S2). The map spanned 3607.3 cM with an average interval of 1.15 cM between adjacent markers (Table S2). On average, each chromosome contained 156 markers spanning an average length of 180.4 cM. The chromosomes ranged from 65.8 cM (Chr 16) to 269.2 cM (Chr 15). The shortest chromosome, Chr 16, was most saturated, containing 95 SNP markers with an average marker density of 0.7 cM. Chr 4 had the largest intervals of 2.3 cM between adjacent markers. The longest chromosome, Chr 15, harbored 319 markers, covering 269.2 cM with an average marker interval of only 0.8 cM.

### 2.3. Identification of QTLs for UV-B Resistance

Based on the constructed genetic map, we identified four QTLs underlying UV-B resistance in soybean on Chr 12, 14, 10, and 6 (in order of logarithm of odds [LOD] score), together explaining 20.1% of phenotypic variation (Table 1). The QTL *UVBR12-1* was located at Chr12:7,261,406…7,273,570 and had the highest LOD score of 3.8, explaining 9.5% of the phenotypic variation. The QTLs *UVBR14-1* and *UVBR10-1* on Chr 14 and 16 had LOD scores of 2.2 and 1.1 and explained 5.3% and 2.7% of phenotypic variation, respectively. On Chr 6, a minor QTL for UV-B resistance between markers Chr06:50,843,417 and Chr06:50,873,593 had the lowest *R*^2^ value (2.6%) among all QTLs detected (Table 1). 

**Figure 2:**
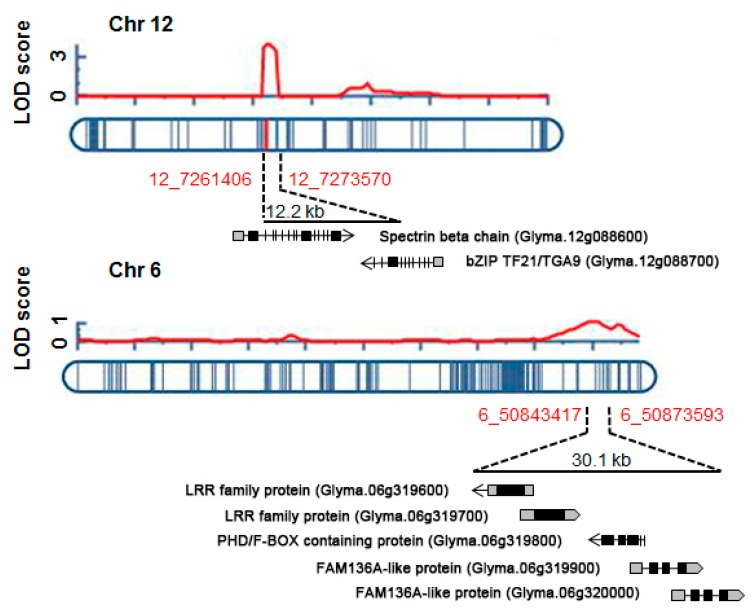
LOD peaks and chromosomal locations of two QTLs *UVBR12-1* and *UVBR6-1* for resistance to high-intensity UV-B irradiation on chromosome (Chr) 12 and Chr 6. Red curves present LOD score distribution of detected QTLs on Chr 12 and Chr 6. These loci contain two and five protein-coding genes, respectively.

We searched for genes located within the four QTLs *UVBR6-1*, *10-1*, *12-1*, and *14-1* according to the physical locations of SNP markers associated with the QTLs (Table 1 and Figure 2). Two genes encoding spectrin beta chain, brain (*SPTBN*, Glyma.12g088600) and bZIP transcription factor21 (*bZIP TF21/TGA9*, Glyma.12g088700) were located within *UVBR12-1* of the 121.6 kb region flanked by the marker positions Chr12:7273570 and Chr12:72761406 (Table 1 and Figure 2). *UVBR6-1* harbored five protein-coding genes anchored in the 30.2 kb region between Chr06:50,843,417 and Chr06:50,873,593 (Table 1 and Figure 2), including two genes (Glyma.06g319600 and Glyma.06g319700) encoding leucine-rich repeat (LRR) family proteins, one gene (Glyma.06g319800) for alfin-like 1, and two genes (Glyma.06g319900 and Glyma.06g320000) encoding family with sequence similarity 136, member A (FAM136A)-like protein (Table 1 and Figure 2). The 1.33 Mb genomic region of *UVBR10-1* (Chr10:41,185,273…Chr10:42,517,624) included 140 genes, and 60 genes were located in the 647.6 kb genomic region corresponding to *UVBR14-1* (Chr14:47,368,499…Chr14:46,720,930) (Table 1 and Table S3).

### 2.4. SNPs and Gene Expression Differences in the QTLs in the Mapping Parents

**Figure 3:**
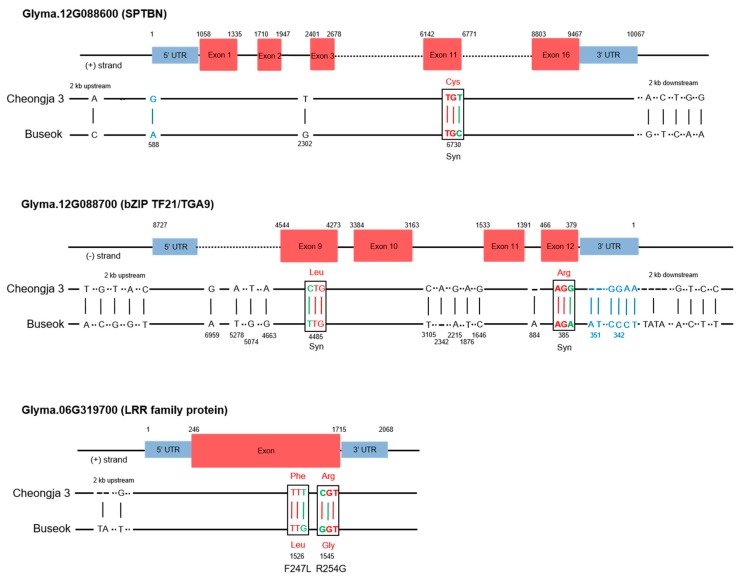
Structures of three genes encoding SPTBN (Glyma.12G088600), bZIP TF21/TGA9 (Glyma.12G088700), and LRR family protein (Glyma.06G319700), and single nucleotide polymorphisms (SNPs) in the mapping parents Cheongja and Buseok. Exons and untranslated regions (UTRs) are indicated by red- and blue-filled boxes, respectively. SNP positions, nucleotide replacements, and amino acid substitutions between Cheongja 3 and Buseok are presented. One and two SNPs in exons of *SPTBN* (Glyma.12G088600) and *bZIP TF21/TGA9* (Glyma.12G088700), respectively, were synonymous. The SNPs T/G and C/G in exons of Glyma.06G319700 (LRR family protein) caused amino acid replacements of Phe to Leu and Arg to Gly at the 247th and 254th residues, respectively.

To identify candidate genes in the QTLs associated with UV-B resistance, we investigated SNPs in two and five genes within the two UV-B resistance QTLs *UVBR12-1* and *UVBR6-1*, respectively, by a sequence analysis of the parental genotypes Cheongja 3 and Buseok (Table). In *UVBR12-1*, the two genes *SPTBN* (Glyma.12G088600) and *bZIP TF21/TGA9* (Glyma.12G088700) had nine and 25 SNPs between Cheongja 3 and Buseok, respectively (Table S4). In *SPTBN* (Glyma.12G088600), we detected one and five SNPs in the up- and downstream regions, respectively. We found three additional SNPs in the genic regions of *SPTBN*, one of which was a synonymous SNP in an exon (Figure 3). Among 25 total SNPs in *bZIP TF21/TGA9* (Glyma.12G088700), we found 15 SNPs in the genic region, consisting of three in 3′UTR, 10 in introns and two in exons (Figure 3 and Table S5). Each of the 2 kb up- and downstream regions of *bZIP TF21/TGA9* possessed five SNPs. Two SNPs in the coding sequence of *bZIP TF21/TGA9* were synonymous (Figure 3).

Among five genes within *UVBR6-1*, three genes had nucleotide differences between Cheongja 3 and Buseok (Tables S4 and S5). In Glyma.06g319700, encoding a LRR family protein, we detected two nonsynonymous missense mutations in exons and two SNPs in the 2 kb upstream region (Figure 3). In particular, we discovered amino acid changes from Phe to Leu and Arg to Gly at positions 247 and 254 of Glyma.06g319700, respectively. The two genes Glyma.06g319900 and Glyma.06g320000 (encoding FAM136A-like proteins) had eight and five SNPs in non-coding regions, respectively (Table S5).

Among five genes within *UVBR6-1*, three genes had nucleotide differences between Cheongja 3 and Buseok (Tables S4 and S5). In Glyma.06g319700, encoding a LRR family protein, we detected two nonsynonymous missense mutations in exons and two SNPs in the 2 kb upstream region (Figure 3). In particular, we discovered amino acid changes from Phe to Leu and Arg to Gly at positions 247 and 254 of Glyma.06g319700, respectively. The two genes Glyma.06g319900 and Glyma.06g320000 (encoding FAM136A-like proteins) had eight and five SNPs in non-coding regions, respectively (Table S5).

**Figure 4:**
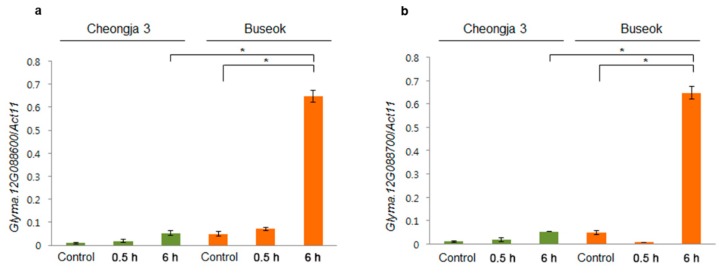
Expression levels of *SPTBN* (Glyma.12G088600) (**a**) and *bZIP TF21/TGA9* (Glyma.12G088700) (**b**) in Cheongja 3 (green) and Buseok (orange). Y-axis represents the relative transcript abundance determined by qRT-PCR. Control, 0.5, and 6 h on the X-axis refer to 0, 0.5, and 6 h UV-B irradiation, respectively. Error bars represent the standard error for three independent replicates. *, significant at *p* < 0.05.

We further investigated transcriptional differences of *SPTBN* (Glyma.12G088600) and *bZIP TF21/TGA9* (Glyma.12G08870) on *UVBR12-1* between Cheongja 3 and Buseok by qRT-PCR (Figure 4) owing to the lack of protein sequence differences between the mapping parents (Figure 3). Both of the genes were upregulated in Buseok subjected to 6 h of UV-B treatment compared with levels in the control. Relative to UV-B-sensitive Cheongja 3, Buseok showed significantly higher expression levels of the two genes in response to 6 h of UV-B exposure.

### 2.5. Comparisons of UV-B Stress Resistance QTLs with Known Stress-Related QTLs Based on Synteny

**Figure 5:**
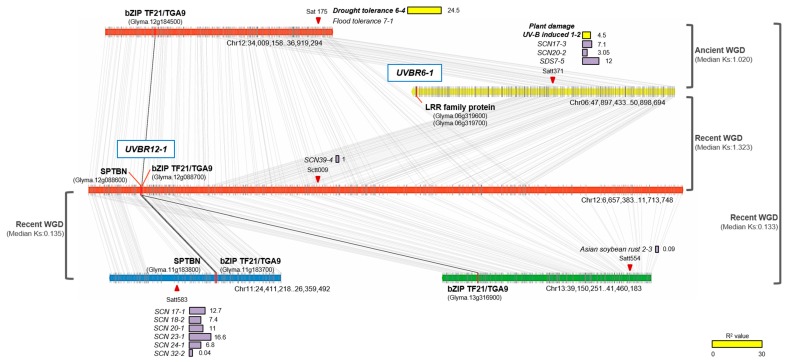
Syntenic conservation of two genomic regions of *UVBR12-1* and *UVBR6-1* and comparisons with other known stress-related QTL regions. The genomic region with *UVBR12-1* (Chr12:6,657,383…11,713,748) had a duplicated block on the same Chr 12 (Chr12:34,009,158...36,919,294) and three additional homeologous regions on Chr 6, 11, and 13. Whole genome duplication (WGD) events producing each pair of syntenic blocks are defined as recent or ancient based on median Ks values. Among two genes in *UVBR12-1*, *bZIP TF21/TGA9* was retained in all duplicated regions but *SPTBN* was only retained in the homeologous block on Chr 11 and was lost in the other blocks. Yellow and purple bars indicate phenotypic R2 values of known QTLs related to abiotic and biotic stress, respectively. SPTBN, spectra beta chain, brain; bZIP TF21/TGA9, bZIP transcription factor 21/TGACG motif-binding 9; SCN, Reaction to *Heterodera glycines*; SDS, Reaction to *Fusarium solani* f. sp. *glycines* infection.

The QTL with the highest LOD, *UVBR12-1*, on Chr 12 was located near *SCN39-4*, a QTL for the reaction to the soybean cyst nematode (SCN; *Heterodera glycines*) linked to Sctt009 (Table 1). The genomic region (Chr12:6,657,383…11,713,748) harboring *UVBR12-1* and *SCN39-4* had a duplicated block on the same chromosome (Chr12:34,009,158…36,919,294), carrying QTLs for drought and flood tolerance (*Drought tolerance 6-4* and *Flood tolerance 7-1*, respectively) linked to Sat 175 (Figure 5). Three homeologous blocks that show syntenic relationships with the two duplicated regions on Chr 12 were also detected on Chr 6, 11, and 13. The homeologous region (Chr11:24,411,218…26,359,492) on Chr 11 with a median Ks value of 0.135 probably resulted from the recent whole genome duplication (WGD) event 13 million years ago [13] and only had collinearity with the beginning of the genomic region (including only *UVBR12-1*) on Chr 12. This syntenic block contained six QTLs for SCN resistance, *SCN 17-1*, *18-2*, *20-1*, *23-1*, *24-1*, and *32-2*, associated with Satt583. On Chr 13, the marker Satt554, associated with the QTL *Asian soybean rust 2-3* for resistance to Asian soybean rust, resided in another duplicated region (Chr13:39,150,251…41,460,183) resulting from the recent WGD (median Ks, 0.133). In *UVBR12-1*, *bZIP TF21/TGA9* was retained in all three duplicated regions but *SPTBN* was only retained in the homeologous block on Chr 11 and was lost in the other blocks (Figure 5). On Chr 6, the remaining duplicated block (Chr06:47,897,433…50,898,694) was mainly conserved with the middle part of the genomic region of *UVBR12-1*. This block with the UV-B resistance QTL *UVBR6-1* included another UV-B-related QTL (*Plant damage UV-B induced 1-2*) as well as biotic stress-related QTLs, including *SCN 17-3* and *20-2*, and *SDS 7-5* for sudden death syndrome resistance (reaction to *Fusarium solani* f. sp. *glycines*), which were associated with Satt371.

The UV-B resistance QTL *UVR14-1* on Chr 14 was mapped near three QTLs for iron efficiency, *Fe-effect 3-2*, *9-2*, and *10-3* (Table 1). On Chr 10, *UVR10-1* was co-localized with several QTLs related to biotic as well as abiotic stresses, including *Flood tolerance 4-7*, *Drought tolerance 6-3*, *Sclero* (*Reaction to Sclerotinia sclerotiorum infection*) *2-23*, *4-10*, and *3-18*, and *Phytoph* (*Reaction to Phytophthora sojae infection*) *5-3*.

## 3. Discussion

Crop plants under abiotic stresses, such as drought, salinity, and extreme temperatures, induce common cellular signaling mechanisms associated with osmotic stress, which disrupts homeostasis and alters the ion balance in the cell [14,15,16]. Similarly, high-intensity UV-B radiation above ambient levels induces cellular responses to nonspecific (genotoxic) damage, similar to various stress defense mechanisms but distinct from photomorphogenesis to low-dose UV-B, indicating that it is a complex environmental stressor [17,18,19,20,21,22]. We previously found that, compared with gene expression in UV-B-sensitive Cheongja 3, UV-B-resistant Buseok exhibits the upregulation of genes involved in phosphatidic acid-dependent signaling pathways as well as several downstream pathways, such as ABA signaling, mitogen-activated protein kinase cascades, and reactive oxygen species overproduction [12]. Therefore, high-density UV-B irradiation is a useful stress treatment for molecular biological and genetic studies of genes involved in extensive resistance to multiple abiotic stresses caused by climate change. 

To identify the genetic elements responsible for UV-B resistance by QTL mapping, we developed a RIL population from a cross between UV-B-sensitive Cheongja 3 and UV-B-resistant Buseok. High-intensity UV-B irradiation turns most leaves yellow with red spots in UV-B-sensitive Cheongja 3, resulting in a dramatic loss in aerial dry weight, whereas Buseok retains more healthy leaves [11,12]. Our RIL population showed a wide distribution in the degree of leaf damage caused by UV-B stress (Figure 1). We identified four QTLs associated with UV-B resistance on Chr 12, 14, 10, and 6 (in order of LOD score) in this population, and all LOD scores were less than four (Table 1). Using a different UV-B-resistant soybean genotype, Iksan 10, previous QTL studies based on SSR genotyping and a SoyaSNP assay identified two major loci for UV-B resistance on Chr 19 and 7, respectively [9,10]. These previously reported QTLs do not overlap with our UV-B resistance QTLs. We identified a novel QTL, *UVBR12-1*, with a LOD score of 3.76 on Chr 12, explaining about 10% of phenotypic variation (Table 1). Thus, the genetic determinants of UV-B resistance in the two soybean genotypes Buseok and Iksan 10 are presumed to be different. However, a minor QTL, *UVBR6-1*, on Chr 6 was mapped in the vicinity of a known minor QTL *UV-B induced plant damage 1-2* associated with Satt371 [9] (Table 1 and Figure 5), which was only detected by multiple regression in the previous study [9].

The newly identified UV-B resistance QTLs were co-localized with other known QTLs for resistance to biotic as well as abiotic stresses (Table 1 and Figure 5). These biotic stresses include infections with *H. glycines*, *F. solani* f. sp. *glycines*, *S. sclerotiorum*, and *P. sojae*, and the abiotic stresses include drought, flood, and iron deficiency. Plants subjected to different combinations of multiple stresses show extensive overlap and crosstalk between stress-response signaling pathways, together with specific responses to individual stresses, indicating a high degree of complexity in plant molecular responses to external stresses [23]. Our results indicate that some genetic elements mediating resistance to UV-B stress may be shared with mechanisms underlying responses to other stresses, consistent with previous findings [12,24]. 

We identified candidate genes controlling resistance to high-intensity UV-B irradiation among two genes on *UVBR12-1* and six genes on *UVBR6-1* (Figure 3 and Figure 4). The gene Glyma.12G088600 on *UVBR12-1* is a homolog of *Arabidopsis SPTBN* (AT5G22450), likely encoding a β-spectrin (βI to βV), which are actin-binding proteins in mammals [25]. Though plants, including *Arabidopsis*, lack spectrins or spectrin-like proteins [26,27], sequences with spectrin repeats and N-terminal calponin homology domains for actin binding are present in the *Arabidopsis* genome [28]. Additionally, spectrins or spectrin-like proteins are localized in plant cellular organelles, such as the Golgi apparatus [29], endoplasmic reticulum [30], and nucleus [31], and in the border plasma membrane of elongating plant cells [32,33]. In mammalian cells, actins bound to β-spectrins and other actin-binding proteins, such as Protein 4.1, Adducin, and Dematin, are connected to the junctional complex at the intracellular side of the plasma membrane [34,35,36]; these are implicated in signal targeting as well as the maintenance of cell shape and structure [25,36]. β-Spectrins interact with membrane phosphoinositides (PtdIns) via Pleckstrin homology (PH) domains present in a number of proteins involved in cellular signaling [37,38,39]. Direct evidence for a relationship between the spectrin-based membrane cytoskeleton and plant stress signaling is very limited; however, levels of PtdIns, such as PtdIns4P and PtdIns(4,5)P2, and other phospholipid molecules in plant cells are increased by osmotic stress from salinity and dehydration [40]. Several genes encoding phosphatidylinositol phosphate kinases and phosphatidylinositol-specific phospholipase C involved in phospholipid signaling are upregulated in Buseok after exposure to high-intensity UV-B [12]. In this study, despite a lack of amino acid differences between the mapping parents (Figure 4 and Table S6), elevated *SPTBN* expression in response to UV-B stress was observed in Buseok but not in Cheongja 3 (Figure 4). Further investigations are necessary to characterize the cellular function of SPTBN in soybean using knockout and overexpression mutants.

HY5 (elongated hypocotyl 5) and HYH (HY5 homolog) are bZIP TFs induced by UV-B. They are key components of low-level UV-B photomorphogenic signaling mediated by β-propeller protein and E3 ubiquitin ligase, encoded by *UVR8* (*UV Resistance Locus 8*) and *COP1* (*Constitutively Photomorphogenic 1*), respectively [19,41,42]. The bZIP TF gene Glyma.12g088700 on *UVBR12-1* is an ortholog of *Arabidopsis* bZIP21/TGA9 (AT1G08320), which belongs to Clade IV of the TGACG motif-binding (TGA) protein family and is essential for anther development [43,44]. In a phylogenetic tree of bZIP genes from *G. max* and *A. thaliana*, the bZIP21/TGA9 orthologs Glyma.12g088700 and AT1G08320 belong to a different cluster from that including HY5 (AT5g11260) (Figure S2), corresponding to previous classifications in *A. thaliana* [43]. The expression of Glyma.12g088700 on *UVR12-1* was significantly increased by exposure to high-intensity UV-B in Buseok (Figure 4), but direct evidence for the role of this type of bZIP gene in resistance to abiotic stresses is lacking. bZIP21/TGA9 and TGA10 are only known to be involved in plant pathogen-associated molecular pattern-triggered immunity stimulated by the immunogenic peptide of bacterial flagellin (flg22) [45,46]. 

In *UVBR6-1*, Glyma.06g319700 showed amino acid differences between the mapping parents; the gene is orthologous to the *Arabidopsis* gene AT1G33590, which encodes a LRR family protein. The LRR motif plays a central role in recognizing different pathogen-associated molecules in the innate host defense of plants and animals [47]. Recent studies have shown that several genes encoding LRR-containing proteins are involved in the abiotic stress response [48], including *LP2* (*LEAF PANICLE 2*), *TaPRK2697* (*Triticum aestivum PROTEIN OF RECEPTOR KINASES 26697*), *AtPXL1* (*Arabidopsis thaliana PHLOEM INTERCALATED WITH XYLEM-LIKE 1*), and *LRR-RLK-VIII* (*LEUCINE-RICH REPEAT RECEPTOR-LIKE KINASE VIII*), which are upregulated by drought, salt, cold, and toxic metals, respectively, in diverse plant species [49,50,51,52].

Three candidate genes responsible for UV-B resistance in soybean were identified from two QTLs, *UVBR12-1* and *UVBR6-1*. These genes encode SPTBN, bZIP TF21/TGA9, and a LRR family protein and are likely involved in stress defense signaling; they should be experimentally verified using overexpression or knockout mutants. Further functional studies will improve our understanding of UV-B stress defense mechanisms. Furthermore, our results provide powerful genetic tools for more efficient and precise breeding programs aimed at the development of highly adaptable soybean cultivars under various abiotic stresses caused by global climate change.

## 4. Materials and Methods 

### 4.1. Plant Materials and DNA Extraction

Two soybean genotypes were used as parents to develop a RIL population for genetic map construction and the QTL analysis. Buseok, a paternal line, is a UV-B-resistant genotype, and Cheongja 3, a maternal line, is a UV-B-sensitive genotype [11,12]. Artificial crossing was performed in summer 2012, and 176 F_6_ RILs were generated from F_2_ seeds using the single seed descent method from winter 2012 to spring 2016. Healthy young leaves from two parental genotypes and RILs were collected, and high-quality genomic DNA was extracted using the GeneAll^®^ Exgene Plant SV Kit (GeneAll Biotechnology, Seoul, Korea). DNA quality was assessed by the 260/280 nm ratio using a Nanodrop 3000 spectrometer (Thermo Scientific, Wilmington, DE, USA). DNA was quantified using the Invitrogen Quant-iT PicoGreen^®^ dsDNA Assay Kit (Life Technologies, Burlington, ON, Canada) and adjusted to 20 ng/μL.

### 4.2. UV-B Treatment and Phenotypic Evaluation

Soybean seeds of the mapping parents and their RILs were planted (three seeds per pot) in 3 L pots containing a 1:1 mixture of desalinated sand and commercial potting soil (Baroker, Seoul Bio Co., Seoul, Korea) in a greenhouse at the experimental farm of Seoul National University, Suwon, Korea in August 2016. Supplemental UV-B irradiation was used to treat soybean plants at the V3 growth stage two to three weeks after emergence according to a previous study [11]. Supplemental UV-B radiation was applied using G40T10E UV-B lamps (Sankyo Denki, Nagano, Japan) with a mean UV-B intensity of 5.68 ± 0.4 Wm^−2^ at the plant level under the lamps. The plants were exposed to high-intensity UV-B stress for 1 h every day at 11:00 am from 17 August to 20 August. The intensity of 1 h of UV-B irradiation was equivalent to two times 11.5 kJ/m^2^, the daily soybean UV-B biological effective dose [12,53]. Three sets of first and second trifoliate leaves above unifoliate leaves from three plants after UV-B treatment were collected with three replications per line. To investigate leaf color changes caused by high-intensity UV-B exposure, the collected leaf samples were scanned with an EPSON Perfection V33 scanner (Epson Inc., Long Beach, CA, USA) and scanned images were analyzed using the WinDIAS 3 Leaf Image Analysis System (DELTA-T DEVICES LTD., Cambridge, UK). The degree of damage was scored on a scale of 1–10 (where 1 = 0%–10%, 2 = 11%–20%, and up to 10 = 91%–100%; damaged area/healthy area × 100 (%)) and these scores was used in QTL mapping for UV-B resistance. Phenotypic distribution was tested for deviation from normality with the Shapiro–Wilk test.

### 4.3. Genotyping-by-Sequencing

A GBS technique was used to detect SNPs to genotype the RIL population. A GBS library was constructed following the protocol described by [54]. DNAs from the mapping parents and 176 RILs were digested individually with ApeKI, which recognizes a degenerate 5 bp sequence (GCWGC, where W is either A or T). Barcoded adapters were ligated with digested DNA fragments (Table S6), and ligated DNA was amplified with appropriate primers. Then, two separate libraries were constructed by pooling amplified DNA samples of 88 RILs for each library. Single-end sequencing of the GBS libraries was performed on two lanes of an Illumina HiSeq2000 instrument (Illumina Inc., San Diego, CA, USA). 

### 4.4. Sequence Analysis, Genetic Map Construction, and QTL Analysis

Raw GBS reads were mapped against the *G. max* reference genome (Wm82.a2) downloaded from Phytozome (https://phytozome.jgi.doe.gov/pz/portal.html) using Bowtie v2.1 after sequence quality control, such as the removal of barcode, adapter, and ApeKI overhang sequences as well as reads with Phred scores of <15 for at least 80% of bases [55,56]. SNPs were called using in-house python scripts with the following filtering criteria: read depth ≥3, Q value ≥30, and missing error rate ≤10%. All SNPs were deposited in The European Variation Archive (https://www.ebi.ac.uk/eva, PRJEB32685). Using identified SNP markers, a genetic map was constructed with JoinMap 4.1 (https://www.kyazma.nl/index.php/mc.JoinMap). SNP markers were grouped using the Kosambi mapping function, and segregation distortion of individual markers was calculated using the χ^2^ test in JoinMap 4.1. The SNP genotyping data and degrees of leaf damage by UV-B treatment for 176 RILs of Cheongja 3 × Buseok were evaluated using QTL IciMapping v.4.1.0.0, and QTLs for resistance to high-intensity UV-B were identified by inclusive composite interval mapping (https://www.integratedbreeding.net/386/breeding-services/more-software-tools/icimapping). To determine the statistically significant threshold for the LOD score, 1000 permutation test was performed at the 5% significance level. 

### 4.5. SNP Survey and qRT-PCR Analysis of Genes in the QTLs for UV-B Stress Resistance

To investigate SNPs in genes located in the QTLs for UV-B resistance in Cheongja 3 and Buseok, whole genome sequences of these genotypes reported in a previous study were used [11]; the sequences are available from the website of the Crop Genomics Laboratory at Seoul National University (http://plantgenomics.snu.ac.kr/). To detect high-confidence SNPs, the following cut-off values were used: minimum read depth of 5, maximum read depth of 20, and Phred-scaled probability score above 30. A maximum read depth of 20 was applied to remove false positive SNP calls that may result from duplicated or repetitive sequences. The SNP positions were determined based on the Glyma 2.0 gene models (Wm82.a2.v1). To compare gene expression levels within the QTL *UVBR12-1* for UV-B resistance between Cheongja 3 and Buseok, gene-specific primers were designed for qRT-PCR using Primer3 (http://bioinfo.ut.ee/primer3-0.4.0/) (Table S7). Total RNA from each sample for control, 0.5 h, and 6 h UV-B treatments in Cheongja 3 and Buseok was used to synthesize cDNA using a Bio-Rad iScript™ cDNA Synthesis Kit (Hercules, CA, USA). UV-B treatments for qRT-PCR analysis followed as described in the previous RNA-seq study [12]: 0.5 h exposure (equivalent to 11.5 KJ/m^2^ soybean UV-B_BE_) induced photomorphogenic (non-damaging) cellular response to low-dose UV-B radiation, while 6 h exposure (12-times higher than daily UV-B_BE_) resulted in nonspecific (genotoxic) damage induced by abiotic stress. qRT-PCR was performed using the synthesized cDNA as a template and a Bio-Rad iQ™ SYBR Green Supermix Kit on a LightCycler^®^ 480 (Roche Diagnostics, Laval, QC, Canada). The qRT-PCR mixture in a total volume of 20 μL contained 100 ng of cDNA, each primer at 300 μM, 8 μL of sterile water, and 10 μL of Bio-Rad iQ™ SYBR Green Supermix. The amplification conditions were as follows: 5 min of denaturation at 95 °C followed by 40 cycles of 95 °C for 10 s and 60 °C for 1 min. The samples were analyzed in triplicate, and actin was used as a reference gene for the normalization of target gene expression in soybean. Data were analyzed based on the stable expression level of the reference gene according to the method of Livak and Schmittgen [57].

### 4.6. Co-Localization of Stress-Related QTLs with UV-B Resistance QTLs

Abiotic and biotic stress-related QTLs for soybean were obtained from the SoyBase website (http://soybase.org/). The chromosomal positions of QTLs on soybean chromosomes were determined using marker information from version 4.0 of the soybean map from SoyBase [58]. Syntenic blocks in the soybean reference genome sequence were analyzed using MCScanX with default parameters [59], and syntenic blocks overlapping with genomic regions of approximately 2 to 5 Mbp encompassing SNP markers associated with UV-B resistance were identified.

## Figures and Tables

**Table 1:** Quantitative trait loci (QTLs) for UV-B stress resistance identified by inclusive composite interval mapping in 176 RILs derived from Cheongja 3 × Buseok.

Locus	Left Marker	Right Marker	Position ^a^ (cM)	LOD ^b^	Add ^c^	*R*^2 d^ (%)	No. of Genes ^e^	Known Stress-Related QTLs ^f^
*UVBR12-1*	Chr12:7261406	Chr12:7273570	74	3.8	-0.4	9.5	2	*Drought tolerance 6-4* *SCN 39-4*
*UVBR14-1*	Chr14:46720930	Chr14:47368499	189	2.2	0.3	5.3	60	*Fe-effect 3-2, 9-2, 10-3*
*UVBR10-1*	Chr10:41185273	Chr10:42517624	160	1.1	-0.2	2.7	142	*Flood tolerance 4-7* *Drought tolerance 6-3* *Sclero 2-23, 4-10, 3-18* *Phytoph 5-3*
*UVBR6-1*	Chr06:50843417	Chr06:50873593	38	1.1	0.2	2.6	5	*Plant damage, UV-B induced 1-2*

^a^ Genetic position of a QTL peak in the linkage map constructed in the present study. ^b^ Maximum-likelihood logarithm of odds (LOD) score for the individual QTL. ^c^ Allelic effect. ^d^ Percent of phenotypic variance explained by the QTL. ^e^ Number of protein-coding genes within marker intervals on the basis of G. max gene models ver. 1.1. ^f^ Known stress-related QTLs within 2 Mb surrounding the QTLs identified in this study.

## Supplementary Materials

Supplementary materials can be found at https://www.mdpi.com/1422-0067/20/13/3287/s1.

## Abbreviations

UV-B	Ultraviolet-B
QTL	Quantitative trait locus
RIL	Recombinant inbred line
LOD	Logarithm of odds
GBS	Genotyping-by-sequencing
SNP	Single nucleotide polymorphism
WGD	Whole genome duplication
SCN	Soybean cyst nematode
SPTBN	Spectrin beta chain, brain
bZIP TF21/TGA9	bZIP transcription factor21/TGACC motif-binding 9
LRR	Leucine-rich repeat
