# Supplementary material for: QTL Analysis of Resistance to High-Intensity UV-B Irradiation in Soybean (Glycine max [L.] Merr.)
Source: Int J Mol Sci. 2019 Jul 4;20(13):3287. doi: 10.3390/ijms20133287 (PMC6651677; doi:10.3390/ijms20133287)
Supplement: Supplementary file 1 [file ijms-20-03287-s001.pdf]

Supplementary Tables and Figures

Supplementary Table 1. Statistic summary of GBS sequencing and SNP data.

	Library 1	Library 2
No. of lines	88	88
No. of raw GBS reads	280,550,845	282,130,604
No. of high-quality clean reads	220,247,631	220,247,626
Average of reads per line	3,188,077	3,206,029
Maximum	8,398,367	10,657,712
Minimum	1,410,429	1,147,211
Total no. of SNPs	271,254	
No. of filtered SNPs	4,604	

Supplementary Table 2. Marker number and average marker interval by chromosome of the soybean genetic linkage map from the Cheongja 3 x Buseok population.

Linkage group	Number of markers	Length (cM)	Avg. marker distance (cM)
Chr01	106	163.5	1.5
Chr02	132	101.9	0.8
Chr03	114	215.1	1.9
Chr04	61	139.3	2.3
Chr05	59	121.9	2.1
Chr06	250	248.5	1.0
Chr07	105	78.9	0.8
Chr08	166	270.0	1.6
Chr09	166	175.3	1.1
Chr10	217	247.4	1.1
Chr11	64	93.8	0.5
Chr12	106	176.3	1.7
Chr13	195	164.7	0.8
Chr14	198	213.0	1.1
Chr15	319	269.2	0.8
Chr16	95	65.8	0.7
Chr17	227	235.0	1.0
Chr18	310	255.3	0.8
Chr19	83	162.2	2.0
Chr20	163	210.2	1.3
Total	3,136	3607.3	1.2

Supplementary Table 3. List of two, five, and 60 genes located in three QTLs *UVBR12-1*, *UVBR6-1*, and *UVBR14-1*, respectively.

***G.max* ID**	**Gene position**	**Gene annotation**	***A.Thaliana* homolog**
Glyma.12g088600	Chr12:7254584..7264767	Unknown protein	AT5G22450.1
Glyma.12g088700	Chr12:7269086..7277812	bZIP transcription factor family protein (bZIP21/TGA9)	AT1G08320.1
Glyma.06g319600	Chr06:50850357..50851530	Leucine-rich repeat (LRR) family protein	AT1G33590.1
Glyma.06g319700	Chr06:50856253..50858320	Leucine-rich repeat (LRR) family protein	AT1G33590.1
Glyma.06g319800	Chr06:50861494..50863853	alfin-like 1	AT5G05610.1
Glyma.06g319900	Chr06:50865413..50868583	FAM136A-like protein	AT2G31725.1
Glyma.06g320000	Chr06:50870241..50873816	FAM136A-like protein	AT2G31725.1
Glyma.14g201800	Chr14:46728613..46733836	Peroxidase superfamily protein	AT5G58400.1
Glyma.14g201900	Chr14:46736415..46739060	Defender against death (DAD family) protein (ATDAD1)	AT1G32210.1
Glyma.14g202000	Chr14:46741879..46743234	RING/U-box superfamily protein with ARM repeat domain	AT2G23140.1
Glyma.14g202100	Chr14:46745358..46746830	Tyrosyl-tRNA synthetase, class Ib, bacterial/mitochondrial	AT3G02660.1
Glyma.14g202200	Chr14:46749403..46751364	Tetratricopeptide repeat (TPR)-like superfamily protein	AT1G12300.1
Glyma.14g202300	Chr14:46754911..46760152	LOB domain-containing protein 13 (LBD13)	AT2G30340.1
Glyma.14g202400	Chr14:46768802..46772377	Exostosin family protein	AT1G21480.1
Glyma.14g202500	Chr14:46773824..46775761	Exostosin family protein	AT1G21480.1
Glyma.14g202600	Chr14:46775998..46777307	Duplicated homeodomain-like superfamily protein	AT3G11280.1
Glyma.14g202700	Chr14:46777725..46778009	CBL-interacting protein kinase 2 (CIPK2)	AT5G07070.1
Glyma.14g202800	Chr14:46779014..46779719	Methyltransferase-related protein	AT5G58375.1
Glyma.14g202900	Chr14:46782700..46783789	allergen-related (CHAL, EPFL6)	AT2G30370.1
Glyma.14g203000	Chr14:46787303..46790554	SOS3-interacting protein 4 (CIPK11,PKS5,SIP4,SNRK3.22)	AT2G30360.1
Glyma.14g203100	Chr14:46803543..46806680	allergen-related (CHAL, EPFL6)	AT2G30370.1
Glyma.14g203200	Chr14:46804365..46804565	Unknown protein	
Glyma.14g203300	Chr14:46813126..46814258	Unknown protein	
Glyma.14g203400	Chr14:46818113..46820666	Unknown protein	
Glyma.14g203500	Chr14:46821151..46822027	ovate family protein 17 (ATOFP17,OFP17)	AT2G30395.1
Glyma.14g203600	Chr14:46826523..46827888	ovate family protein 3 (ATOFP3,OFP3)	AT5G58360.1
Glyma.14g203700	Chr14:46828582..46834138	with no lysine (K) kinase 4 (WNK4, ZIK2)	AT5G58350.1
Glyma.14g203800	Chr14:46841491..46853116	PAP/OAS1 substrate-binding domain superfamily	AT3G51620.2
Glyma.14g203900	Chr14:46856580..46859641	expansin A8 (ATEXP8,ATEXPA8,ATHEXP ALPHA 1.11,EXP8,EXPA8)	AT2G40610.1
Glyma.14g204000	Chr14:46883204..46883614	Unknown protein	
Glyma.14g204100	Chr14:46886862..46890243	Basic-leucine zipper (bZIP) transcription factor family protein	AT2G40620.1
Glyma.14g204200	Chr14:46893813..46897703	extra-large G-protein 1	AT2G23460.1
Glyma.14g204300	Chr14:46909226..46909477	AGC (cAMP-dependent, cGMP-dependent and protein kinase C) kinase family protein	AT2G20470.1
Glyma.14g204400	Chr14:46936057..46943868	high-level expression of sugar-inducible gene 2 (HSI2,VAL1)	AT2G30470.1
Glyma.14g204500	Chr14:46946496..46957734	NB-ARC domain-containing disease resistance protein	AT4G27190.1

Glyma.14g204600	Chr14:46968705..46974585	NB-ARC domain-containing disease resistance protein	AT4G27220.1
Glyma.14g204700	Chr14:46981104..46996696	NB-ARC domain-containing disease resistance protein	AT4G27220.1
Glyma.14g204800	Chr14:46984060..46984741	Unknown protein	
Glyma.14g204900	Chr14:47000358..47001315	NB-ARC domain-containing disease resistance protein	AT4G27220.1
Glyma.14g205000	Chr14:47005574..47019661	NB-ARC domain-containing disease resistance protein	AT4G27220.1
Glyma.14g205100	Chr14:47020646..47026852	Kinase interacting (KIP1-like) family protein	AT5G58320.2
Glyma.14g205200	Chr14:47041931..47046048	cinnamate-4-hydroxylase (ATC4H,C4H,CYP73A5,REF3)	AT2G30490.1
Glyma.14g205300	Chr14:47046209..47056610	NB-ARC domain-containing disease resistance protein	AT4G27220.1
Glyma.14g205400	Chr14:47059088..47060670	Unknown protein	
Glyma.14g205500	Chr14:47061905..47067563	eukaryotic translation initiation factor 2 beta subunit (EIF2 BETA,EMB1401)	AT5G20920.1
Glyma.14g205600	Chr14:47079122..47081495	cytokinin response factor 4 (CRF 4)	AT4G27950.1
Glyma.14g205700	Chr14:47085767..47091016	ubiquitin conjugating enzyme 8 (ATUBC8,UBC8)	AT5G41700.1
Glyma.14g205800	Chr14:47089296..47089496	Unknown protein	
Glyma.14g205900	Chr14:47094145..47095799	Unknown protein	
Glyma.14g206000	Chr14:47096077..47101943	Leucine-rich repeat protein kinase family protein	AT5G58300.1
Glyma.14g206100	Chr14:47121840..47125184	Phototropic-responsive NPH3 family protein (RPT2)	AT2G30520.1
Glyma.14g206200	Chr14:47136840..47148766	Leucine-rich repeat protein kinase family protein	AT1G06840.1
Glyma.14g206300	Chr14:47152323..47167818	Leucine-rich repeat protein kinase family protein	AT1G06840.1
Glyma.14g206400	Chr14:47170359..47171797	Unknown protein	
Glyma.14g206500	Chr14:47181077..47199983	NB-ARC domain-containing disease resistance protein	AT4G27190.1
Glyma.14g206600	Chr14:47200162..47203571	Cation efflux family protein	AT3G58060.1
Glyma.14g206700	Chr14:47204975..47207366	Cytochrome b-c1 complex, subunit 8 protein	AT3G10860.1
Glyma.14g206800	Chr14:47208588..47212843	RNA-binding KH domain-containing protein	AT2G38610.1
Glyma.14g206900	Chr14:47215859..47220940	NB-ARC domain-containing disease resistance protein	AT4G27220.1
Glyma.14g207000	Chr14:47264742..47265649	Yippee family putative zinc-binding protein	AT4G27745.1
Glyma.14g207100	Chr14:47266214..47268996	Tetratricopeptide repeat (TPR)-like superfamily protein	AT5G16860.1
Glyma.14g207200	Chr14:47290893..47309462	ABC transporter of the mitochondrion 3 (ATATM3,ATM3,STA1)	AT5G58270.1
Glyma.14g207300	Chr14:47312711..47319651	Tetratricopeptide repeat (TPR)-like superfamily protein	AT5G51340.1
Glyma.14g207400	Chr14:47325884..47329827	Unknown protein	
Glyma.14g207500	Chr14:47331497..47335155	TRAM, LAG1 and CLN8 (TLC) lipid-sensing domain containing protein	AT4G10360.1
Glyma.14g207600	Chr14:47338112..47341438	Cystathionine beta-synthase (CBS) family protein	AT5G10860.1
Glyma.14g207700	Chr14:47365910..47369153	Thiamine pyrophosphate dependent pyruvate decarboxylase family protein	AT5G17380.1

Supplementary Table 4. Detailed information of SNPs in genes in the two QTLs *UVBR12-1* and *UVBR6-1* between Cheongja 3 and Buseok.

Gene ID	Location	Cheongja 3	Buseok	Variant	Effect
Glyma.12G088600	Chr12:7253257	A	C	2kb upstream gene variant	Modifier
	Chr12:7255288	G	A	5 prime UTR variation	Modifier
	Chr12:7257002	T	G	Intron variant	Modifier
	Chr12:7261430	T	C	synonymous variant	Low
	Chr12:7264812	A	G	2kb downstream gene variant	
	Chr12:7265032	C	T		
	Chr12:7265072	T	C		
	Chr12:7265224	G	A		
	Chr12:7265449	G	A		
Glyma.12G088700	Chr12:7267156	C	T	2kb downstream gene variant	
	Chr12:7267333	C	T		
	Chr12:7267381	T	C		
	Chr12:7268635	G	A		
	Chr12:7268641	CATATATATA	CATATATATATATA		
	Chr12:7269105	A	T	3 prime UTR variation	Modifier
	Chr12:7269114	A	C		
	Chr12:7269134	G	C		
	Chr12:7269427	G	C		
	Chr12:7269446	AATATATATAT	AATATATATATAT		
	Chr12:7269470	G	A	synonymous variant	Low
	Chr12:7269969	GA	GAA	Intron variant	Modifier
	Chr12:7270731	G	C		
	Chr12:7270961	A	T		
	Chr12:7271300	G	A		
	Chr12:7271427	GAAAAAAAA	GAAAAAAA		
	Chr12:7272190	C	T		
	Chr12:7273748	A	G		
	Chr12:7274159	T	G		
	Chr12:7276044	G	A		
	Chr12:7274363	A	T		
	Chr12:7273570	C	T	synonymous variant	Low
	Chr12:7278085	C	T	2kb upstream gene variant	Modifier
	Chr12:7278656	A	G		
	Chr12:7279303	T	G		
	Chr12:7279517	G	C		
	Chr12:7279520	T	A		
Glyma.06G319700	Chr06:50855321	G	T	2kb upstream gene variant	Modifier
	Chr06:50855927	CTATATATATATATA	CTATATATATATATATA		
	Chr06:50857778	T	G	**missense variant**	High
	Chr06:50857797	C	G	**missense variant**	High
Glyma.06G319900	Chr06:50864988	A	C	2kb upstream gene variant	Modifier
	Chr06:50864989	A	T		
	Chr06:50865052	A	G		
	Chr06:50865118	G	A		
	Chr06:50865950	GTTTTTT	GTTTTTTT	Intron variant	Modifier
	Chr06:50868590	T	C	2kb downstream gene variant	Modifier
	Chr06:50868771	T	C		
	Chr06:50869183	G	T		
Glyma.06G320000	Chr06:50871160	T	C	Intron variant	Modifier
	Chr06:50871325	T	C		
	Chr06:50871579	T	C		
	Chr06:50874007	G	A	2kb downstream gene variant	Modifier
	Chr06:50874122	TACAC	TACACAC		

Supplementary Table 5. Summary of SNPs near and/or within genes in *UVBR12-1* and *UVBR6-1*.

Gene ID	Number of SNPs						
	2kb upstream	5'-UTR	CDS	Intron	3'-UTR	2kb downstream	Total
Glyma.12G088600	1	1	1	1		5	9
Glyma.12G088700	5		2	10	3	5	25
Glyma.06G319600							-
Glyma.06G319700	2		2				4
Glyma.06G319800							-
Glyma.06G319900	4			1		3	8
Glyma.06G320000				3		2	5

CDS, coding sequence; UTR, untranslated region

Supplementary Table 6. Barcode sequences used for GBS library construction.

	Barcode	adapter top	adapter bottom
1	TGACGCCA	cacgacgctcttccgatctTGACGCCA	CWGTGGCGTCAagatcggaagagcgtcgtg
2	CAGATA	cacgacgctcttccgatctCAGATA	CWGTATCTGagatcggaagagcgtcgtg
3	CTCC	acactctttccctacacgacgctcttccgatctCTCC	CWGGGAGagatcggaagagcgtcgtgtagggaaagagtgt
4	TAGCGGAT	cacgacgctcttccgatctTAGCGGAT	CWGATCCGCTAagatcggaagagcgtcgtg
5	TATTCGCAT	cacgacgctcttccgatctTATTCGCAT	CWGATGCGAATAagatcggaagagcgtcgtg
6	ATAGAT	cacgacgctcttccgatctATAGAT	CWGATCTATagatcggaagagcgtcgtg
7	CCGAACA	cacgacgctcttccgatctCCGAACA	CWGTGTTCGGagatcggaagagcgtcgtg
8	GGAAGACAT	cacgacgctcttccgatctGGAAGACAT	CWGATGTCTTCCagatcggaagagcgtcgtg
9	GGCTTA	cacgacgctcttccgatctGGCTTA	CWGTAAGCCagatcggaagagcgtcgtg
10	AACGCACATT	cacgacgctcttccgatctAACGCACATT	CWGAATGTGCGTTagatcggaagagcgtcgtg
11	GAGCGACAT	cacgacgctcttccgatctGAGCGACAT	CWGATGTCGCTCagatcggaagagcgtcgtg
12	CCTTGCCATT	cacgacgctcttccgatctCCTTGCCATT	CWGAATGGCAAGGagatcggaagagcgtcgtg
13	GGTATA	cacgacgctcttccgatctGGTATA	CWGTATACCagatcggaagagcgtcgtg
14	TCTTGG	cacgacgctcttccgatctTCTTGG	CWGCCAAGAagatcggaagagcgtcgtg
15	GGTGT	cacgacgctcttccgatctGGTGT	CWGACACCagatcggaagagcgtcgtg
16	GGATA	cacgacgctcttccgatctGGATA	CWGTATCCagatcggaagagcgtcgtg
17	CTAAGCA	cacgacgctcttccgatctCTAAGCA	CWGTGCTTAGagatcggaagagcgtcgtg
18	ATTAT	cacgacgctcttccgatctATTAT	CWGATAATagatcggaagagcgtcgtg
19	GCGCTCA	cacgacgctcttccgatctGCGCTCA	CWGTGAGCGCagatcggaagagcgtcgtg
20	ACTGCGAT	cacgacgctcttccgatctACTGCGAT	CWGATCGCAGTagatcggaagagcgtcgtg
21	TTCGTT	cacgacgctcttccgatctTTCGTT	CWGAACGAAagatcggaagagcgtcgtg
22	ATATAA	cacgacgctcttccgatctATATAA	CWGTTATATagatcggaagagcgtcgtg
23	TGGCAACAGA	cacgacgctcttccgatctTGGCAACAGA	CWGTCTGTTGCCAagatcggaagagcgtcgtg
24	CTCGTCG	cacgacgctcttccgatctCTCGTCG	CWGCGACGAGagatcggaagagcgtcgtg
25	GCCTACCT	cacgacgctcttccgatctGCCTACCT	CWGAGGTAGGCagatcggaagagcgtcgtg
26	CACCA	cacgacgctcttccgatctCACCA	CWGTGGTGagatcggaagagcgtcgtg
27	AATTAG	cacgacgctcttccgatctAATTAG	CWGCTAATTagatcggaagagcgtcgtg
28	GGAACGA	cacgacgctcttccgatctGGAACGA	CWGTCGTTCCagatcggaagagcgtcgtg
29	ACAACT	cacgacgctcttccgatctACAACT	CWGAGTTGTagatcggaagagcgtcgtg
30	ACTGCT	cacgacgctcttccgatctACTGCT	CWGAGCAGTagatcggaagagcgtcgtg
31	CGTGGACAGT	cacgacgctcttccgatctCGTGGACAGT	CWGACTGTCCACGagatcggaagagcgtcgtg
32	TGGCACAGA	cacgacgctcttccgatctTGGCACAGA	CWGTCTGTGCCAagatcggaagagcgtcgtg
33	TGCTT	cacgacgctcttccgatctTGCTT	CWGAAGCAagatcggaagagcgtcgtg
34	GCAAGCCAT	cacgacgctcttccgatctGCAAGCCAT	CWGATGGCTTGCagatcggaagagcgtcgtg
35	CGCACCAATT	cacgacgctcttccgatctCGCACCAATT	CWGAATTGGTGCGagatcggaagagcgtcgtg
36	CTCGCGG	cacgacgctcttccgatctCTCGCGG	CWGCCGCGAGagatcggaagagcgtcgtg
37	AACTGG	cacgacgctcttccgatctAACTGG	CWGCCAGTTagatcggaagagcgtcgtg
38	ATGAGCAA	cacgacgctcttccgatctATGAGCAA	CWGTTGCTCATagatcggaagagcgtcgtg
39	CTTGA	cacgacgctcttccgatctCTTGA	CWGTCAAGagatcggaagagcgtcgtg
40	GCGTCCT	cacgacgctcttccgatctGCGTCCT	CWGAGGACGCagatcggaagagcgtcgtg
41	ACCAGGA	cacgacgctcttccgatctACCAGGA	CWGTCCTGGTagatcggaagagcgtcgtg
42	CCACTCA	cacgacgctcttccgatctCCACTCA	CWGTGAGTGGagatcggaagagcgtcgtg
43	TCACGGAAG	cacgacgctcttccgatctTCACGGAAG	CWGCTTCCGTGAagatcggaagagcgtcgtg
44	TATCA	cacgacgctcttccgatctTATCA	CWGTGATAagatcggaagagcgtcgtg
45	TAGCCAA	cacgacgctcttccgatctTAGCCAA	CWGTTGGCTAagatcggaagagcgtcgtg
46	ATATCGCCA	cacgacgctcttccgatctATATCGCCA	CWGTGGCGATATagatcggaagagcgtcgtg
47	CTCTA	cacgacgctcttccgatctCTCTA	CWGTAGAGagatcggaagagcgtcgtg
48	GGTGCACATT	cacgacgctcttccgatctGGTGCACATT	CWGAATGTGCACCagatcggaagagcgtcgtg

49	CTCTCGCAT	cacgacgctcttccgatctCTCTCGCAT	CWGATGCGAGAGagatcggaagagcgtcgtg
50	CAGAGGT	cacgacgctcttccgatctCAGAGGT	CWGACCTCTGagatcggaagagcgtcgtg
51	GCGTACAAT	cacgacgctcttccgatctGCGTACAAT	CWGATTGTACGCagatcggaagagcgtcgtg
52	CAGA	acactctttccctacacgacgctcttccgatctCAGA	CWGTCTGagatcggaagagcgtcgtgtagggaaagagtgt
53	GTCGCCT	cacgacgctcttccgatctGTCGCCT	CWGAGGCGACagatcggaagagcgtcgtg
54	AATAACCAA	cacgacgctcttccgatctAATAACCAA	CWGTTGGTTATTagatcggaagagcgtcgtg
55	AATGAACGA	cacgacgctcttccgatctAATGAACGA	CWGTCGTTCATTagatcggaagagcgtcgtg
56	CGTCGCCACT	cacgacgctcttccgatctCGTCGCCACT	CWGAGTGGCGACGagatcggaagagcgtcgtg
57	ATGGCAA	cacgacgctcttccgatctATGGCAA	CWGTTGCCATagatcggaagagcgtcgtg
58	GAAGCA	cacgacgctcttccgatctGAAGCA	CWGTGCTTCagatcggaagagcgtcgtg
59	AACGTGCCT	cacgacgctcttccgatctAACGTGCCT	CWGAGGCACGTTagatcggaagagcgtcgtg
60	CCTCG	cacgacgctcttccgatctCCTCG	CWGCGAGGagatcggaagagcgtcgtg
61	CTCAT	cacgacgctcttccgatctCTCAT	CWGATGAGagatcggaagagcgtcgtg
62	ACGGTACT	cacgacgctcttccgatctACGGTACT	CWGAGTACCGTagatcggaagagcgtcgtg
63	GCGCCG	cacgacgctcttccgatctGCGCCG	CWGCGGCGCagatcggaagagcgtcgtg
64	CAAGT	cacgacgctcttccgatctCAAGT	CWGACTTGagatcggaagagcgtcgtg
65	TCCGAG	cacgacgctcttccgatctTCCGAG	CWGCTCGGAagatcggaagagcgtcgtg
66	TAGATGA	cacgacgctcttccgatctTAGATGA	CWGTCATCTAagatcggaagagcgtcgtg
67	TGGCCAG	cacgacgctcttccgatctTGGCCAG	CWGCTGGCCAagatcggaagagcgtcgtg
68	GCACGAT	cacgacgctcttccgatctGCACGAT	CWGATCGTGCagatcggaagagcgtcgtg
69	TTGCTG	cacgacgctcttccgatctTTGCTG	CWGCAGCAAagatcggaagagcgtcgtg
70	CGCAACCAGT	cacgacgctcttccgatctCGCAACCAGT	CWGACTGGTTGCGagatcggaagagcgtcgtg
71	TCACTG	cacgacgctcttccgatctTCACTG	CWGCAGTGAagatcggaagagcgtcgtg
72	ACAGT	cacgacgctcttccgatctACAGT	CWGACTGTagatcggaagagcgtcgtg
73	GGAGTCAAG	cacgacgctcttccgatctGGAGTCAAG	CWGCTTGACTCCagatcggaagagcgtcgtg
74	CGAT	acactctttccctacacgacgctcttccgatctCGAT	CWGATCGagatcggaagagcgtcgtgtagggaaagagtgt
75	CATAT	cacgacgctcttccgatctCATAT	CWGATATGagatcggaagagcgtcgtg
76	GTGACACAT	cacgacgctcttccgatctGTGACACAT	CWGATGTGTCACagatcggaagagcgtcgtg
77	TATGT	cacgacgctcttccgatctTATGT	CWGACATAagatcggaagagcgtcgtg
78	CAGTGCCATT	cacgacgctcttccgatctCAGTGCCATT	CWGAATGGCACTGagatcggaagagcgtcgtg
79	ACAACCAACT	cacgacgctcttccgatctACAACCAACT	CWGAGTTGGTTGTagatcggaagagcgtcgtg
80	TGCAGA	cacgacgctcttccgatctTGCAGA	CWGTCTGCAagatcggaagagcgtcgtg
81	CATCTGCCG	cacgacgctcttccgatctCATCTGCCG	CWGCGGCAGATGagatcggaagagcgtcgtg
82	AGGC	acactctttccctacacgacgctcttccgatctAGGC	CWGGCCTagatcggaagagcgtcgtgtagggaaagagtgt
83	ATCTGT	cacgacgctcttccgatctATCTGT	CWGACAGATagatcggaagagcgtcgtg
84	AAGACGCT	cacgacgctcttccgatctAAGACGCT	CWGAGCGTCTTagatcggaagagcgtcgtg
85	GAATGCAATA	cacgacgctcttccgatctGAATGCAATA	CWGTATTGCATTCagatcggaagagcgtcgtg
86	GATC	acactctttccctacacgacgctcttccgatctGATC	CWGGATCagatcggaagagcgtcgtgtagggaaagagtgt
87	ATCCG	cacgacgctcttccgatctATCCG	CWGCGGATagatcggaagagcgtcgtg
88	CTTAG	cacgacgctcttccgatctCTTAG	CWGCTAAGagatcggaagagcgtcgtg
89	TTATTACAT	cacgacgctcttccgatctTTATTACAT	CWGATGTAATAAagatcggaagagcgtcgtg
90	GCCAACAAGA	cacgacgctcttccgatctGCCAACAAGA	CWGTCTTGTTGGCagatcggaagagcgtcgtg
91	TGCCGCAT	cacgacgctcttccgatctTGCCGCAT	CWGATGCGGCAagatcggaagagcgtcgtg
92	CGTGTCA	cacgacgctcttccgatctCGTGTCA	CWGTGACACGagatcggaagagcgtcgtg
93	CAACCACACA	cacgacgctcttccgatctCAACCACACA	CWGTGTGTGGTTGagatcggaagagcgtcgtg
94	GCTCCGA	cacgacgctcttccgatctGCTCCGA	CWGTCGGAGCagatcggaagagcgtcgtg
95	TCAGAGAT	cacgacgctcttccgatctTCAGAGAT	CWGATCTCTGAagatcggaagagcgtcgtg
96	CGTTCA	cacgacgctcttccgatctCGTTCA	CWGTGAACGagatcggaagagcgtcgtg

Supplementary Table 7. Primer sequences used for qRT-PCR analysis of genes located in the QTL *UVBR12-1* for UV-B stress resistance.

Gene ID	Gene description	Forward sequence (5'-3')	Reverse sequence (5'-3')	product size (bp)
Glyma.12g088600	Spectrin beta chain, brain (SPTBN)	TCAATGGTGGTGTCAGTGGT	ATTCCTCTCCCTCTCGCTTC	98
Glyma.12g088700	bZIP TF21/TGA9	GGCAATTGTTCTGCAAGGAA	AGGGGAACATTTCCAGTGTG	98
Glyma.18g290800	Actin11	CGGTGGTTCTATCTTGGCATC	GTCTTTCGCTTCAATAACCCTA	142

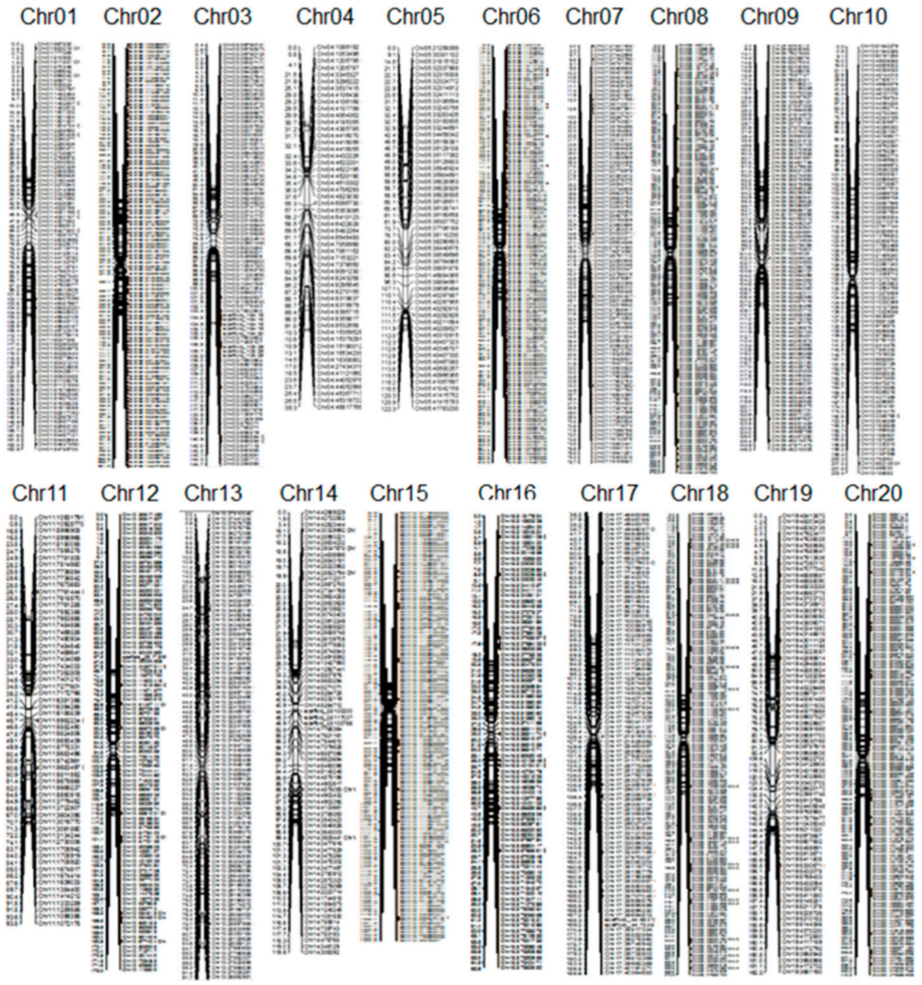

Supplementary Figure 1. Genetic linkage map including 3,136 SNP markers from the Cheongja 3 x Buseok population.

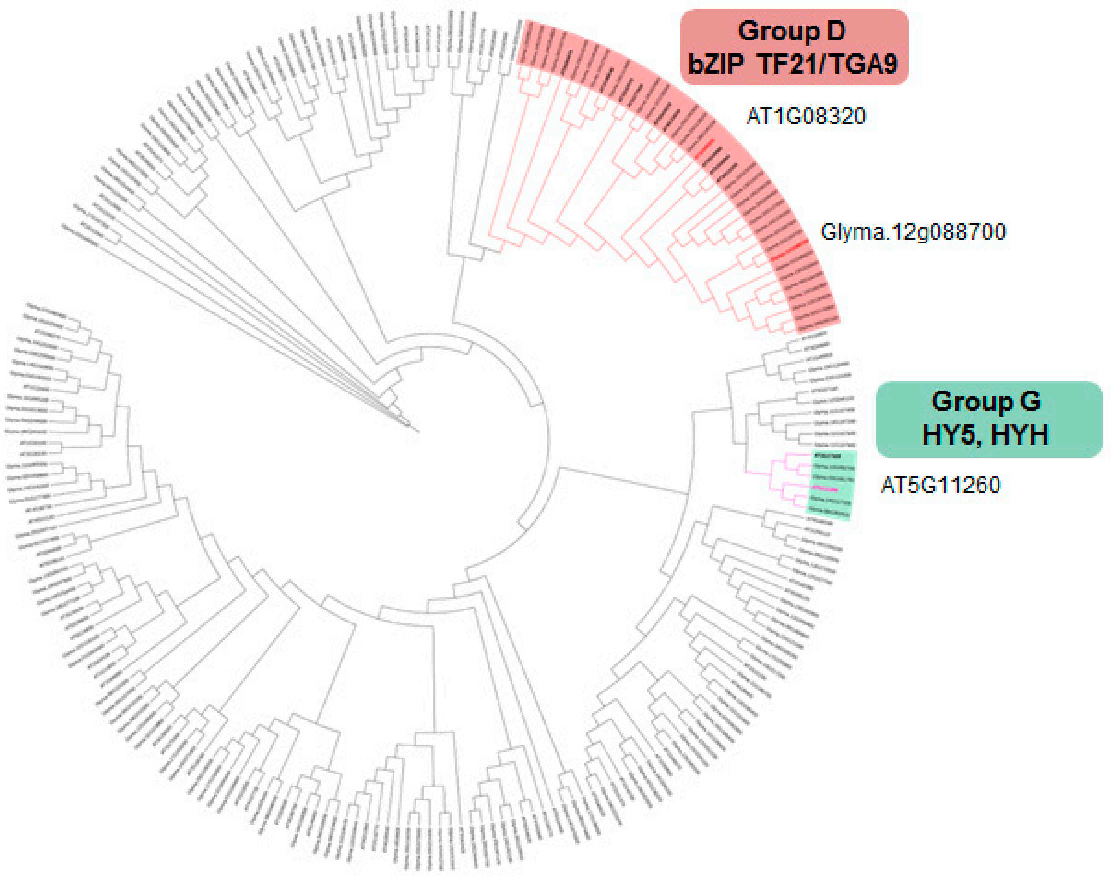

Supplementary Figure 2. Phylogenetic tree of bZIP transcription factors from *G. max* and *A. thaliana*. The bZIP21/TGA9 orthologs Glyma.12g088700 and AT1G08320 belong to a different cluster from that including HY5 (AT5g11260).
